# Getting real about synthetic data ethics: Are AI ethics principles a good starting point for synthetic data ethics?

**DOI:** 10.1038/s44319-024-00101-0

**Published:** 2024-02-22

**Authors:** Danielle Shanley, Joshi Hogenboom, Flora Lysen, Leonard Wee, Aiara Lobo Gomes, Andre Dekker, Darian Meacham

**Affiliations:** Maastricht University, Maastricht, The Netherlands

**Keywords:** Computational Biology, Economics, Law & Politics

## Abstract

Synthetic data promises to be a viable alternative when data collection and data sharing may not be feasible or cost effective, but it raises distinct ethical issue that merit serious consideration.

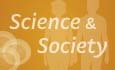

The creation of synthetic data (SD)—‘artificial’ data generated specifically with the goal of resembling ‘real-world’ data in order to replace or reduce the need for ‘real’ data—is booming.

By some estimations, SD “will completely overshadow real data in artificial intelligence (AI) models” by 2030 (Linden, 2022). SD promises to be a viable alternative when data collection and data sharing may not be feasible or cost-effective. However, the increasingly prevalent use of SD for training and benchmarking in AI development is generating novel and pertinent ethical concerns that should be considered by developers and users of synthetic datasets and AI applications (on which they are trained). Here, we provide an overview of issues arising from the rapid growth in SD generation and use to encourage further reflection and investigation within the research community.

## The promise and peril of synthetic data use

In the health, medical and life sciences, the use of SD is far from novel. Its use is instead a standard practice, for example, for the imputation of missing data, in simulations of biological systems or images using deterministic and stochastic approaches, and in the generation of entirely probabilistic data through Bayesian Networks (Gonzales et al, 2023). However, the increasing sophistication in generating SD (for example, through deep learning applications, Sun et al, 2023) make it an increasingly versatile technology. It now promises to both enhance privacy—as entirely generated SD implies the absence of identifiable information of ‘real’ individuals—and potentially mitigate existing biases through deliberately diversifying ‘real’ datasets. SD is also seen as a remedy to the pressing issue of limited sample size through data augmentation, making it especially interesting for technologies that demand large datasets, specifically in AI model development.

“SD is also seen as a remedy to the pressing issue of limited sample size through data augmentation, making it especially interesting for technologies that demand large datasets…”

There is no doubt that the increasing generation and use of SD holds much promise for different areas. By way of example, Gonzales et al, (2023) identified seven possible use cases in the healthcare context: “(a) simulation and prediction research, (b) hypothesis, methods, and algorithm testing, (c) epidemiology/public health research, (d) health IT development, (e) education and training, (f) public release of datasets, and (g) linking data”. They also suggest that datasets containing synthetic data demonstrate “varying degrees of utility for research, education, and software development”. However, the potential disadvantages and dangers of its use and especially its overuse have yet to be fully considered. While questions regarding the ethicality and reliability of using a proxy of reality are not new, SD adds an extra dimension owing to its complexity, as well as to the foreseen scale of its use.

Nonetheless, SD has already been introduced in AI model development, ranging from training and validation using SD resembling probabilistic or stochastic data (Jacques et al, 2021), to models that were fully developed using deterministic SD (Yao et al, 2020). Technical challenges emerging from these recent developments have been discussed in the literature (Achuthan et al, 2022), but ethical challenges, for example regarding possible biases in SD, are no less pertinent than the technical ones that currently dominate the conversation. This is especially relevant for SD that is generated from real data. Augmenting a dataset for the purpose of AI model development can indeed increase sample size, yet it can equally augment the inherent biases and thereby exacerbate an already poor external validity of a model. In medicine, for example, some have already expressed concerns about an overreliance on SD leading to an insufficient resemblance with ‘real’ world situations (e.g., Strickland, 2019). Moreover, despite increasing interest in SD, no legal or ethical frameworks are in place to regulate its utilisation. For example, who retains ownership of SD generated by public resources (D’Amico et al, 2023), and what can this SD lawfully and reliably be used for?

“… despite increasing interest in SD, no legal or ethical frameworks are in place to regulate its utilisation.”

We suggest that the burgeoning use of SD brings with it two key risks. The first is that issues specific to the use of SD might get lost within broader discussions regarding AI and data ethics. Though many implications of SD may look similar to other forms of data use, SD presents sufficiently unique questions and challenges that it warrants more fine-grained attention. The second risk is that positive claims about the value of SD and the ways in which it potentially mitigates certain concerns—such as regarding privacy and data scarcity—might overshadow the possible negative consequences of its use. Insofar as SD is qualitatively different, we argue that we cannot evaluate synthetic datasets in the same way that we would other forms of data.

As we will outline below, the use of SD raises a variety of ethical questions. Now is the time to start thinking about these issues before practices and patterns of use become too entrenched. We sketch a number of preliminary concerns and questions that we see emerging with regard to the possible use(s) of SD. We have organised these around the five ethical principles most prevalent in responsible AI guidelines: responsibility, non-maleficence, privacy, transparency, and justice and fairness (Jobin et al, 2019). Our overview is meant as a springboard to start a discussion about potential concerns and areas of investigation and not meant as a rigid categorisation as many issues pertain to more than one ethical principle. We conclude by drawing attention to key questions that we believe demonstrate the need for further study and reflection on the possible future use(s) of SD.

## Responsibility

Who gets to decide when and for what the use of SD is justified? When is ‘real’ data needed and when is it appropriate to settle for (partial) SD? If the use of SD means taking into account additional considerations during the decision-making process, does its use therefore imply new or different responsibilities for those involved in the AI supply chain? What does its use(s) mean for the roles, responsibilities and decision-making processes of those involved in its generation and use?

In ethical AI guidelines, responsibility in creating and using AI is often associated with “acting with integrity”, with “clarifying the attribution of responsibility and legal liability”, and with “harm reduction” (Jobin et al, 2019). However, key differences emerge concerning which actors are deemed responsible and accountable, from “developers” and “designers” to “institutions” and “industry”. As the role of SD in training, testing and evaluating AI models looks set to increase, it is important to identify how and in what ways it might transform existing ideas about what constitutes ‘responsible AI’. Over time, the use of SD will recurrently influence future data analysis because synthetically generated data will be gradually absorbed into new datasets (see ‘Transparency’). As a consequence, responsible use of SD also means thinking about how a specific use of SD in one instance may influence a much larger data landscape over time and at scale. Accordingly, accountability needs to pertain to a longer time span and involve new ways of tracing the use and effects of SD data—and being accountable for traceability.

## Non-maleficence

What will the gap between the ‘real’ world the AI operates in and the synthetic world it was trained on look like? What means and measures can we use to delineate that gap? And what vocabulary can we use to make sense of data that is not collected in the ‘real’ world in terms of its status vis-à-vis knowledge or truth claims? If the assumption is that synthetic datasets are not only ‘bigger’ and ‘cheaper’, but ‘risk-free’, the likelihood for their uncritical adoption seems high. What is the potential for intentional and unintentional misuse of SD?

There is an argument for using SD to correct the bias of AI systems trained on unrepresentative data, for example. In the case of facial recognition software, SD could be utilised in order to create more diverse datasets. As Joy Buolamwini states, “training sets do not just materialise out of nowhere. We can create them. So there’s an opportunity to create full-spectrum training sets that reflect a richer portrait of humanity” (https://broutonlab.com/blog/ai-bias-solved-with-synthetic-data-generation/). However, we also need to be aware of an overreliance on technological fixes. Using SD to make unrepresentative data representative presents new and as yet unknown challenges, not least simply serving to mask real-world inequities. The rapid uptake of machine-generated information combined with their propensity towards factual inaccuracy has also been shown to increase the production and spread of misinformation (Hanley and Durumeric, 2023).

Across AI ethics guidelines, references to non-maleficence “encompass general calls for safety and security or state that AI should never cause foreseeable or unintentional harm” (Jobin et al, 2019). Researchers and regulators should be wary of claims that SD insulates algorithms from violations of privacy, discrimination or bias by providing datasets that are “supposedly varied, unbiased, balanced, representative, and therefore risk-free” (Jacobsen, 2023). Creators of SD still have to make decisions about what to include and exclude. Whilst synthetic variability may appear to decrease bias on the surface, it simultaneously serves to amplify particular ways of seeing the world. An overreliance on SD as a tool for reducing risk of harm may also serve to perpetuate the notion that machine-learning algorithms can be neutral, objective and controllable.

“An overreliance on SD as a tool for reducing risk of harm may also serve to perpetuate the notion that machine-learning algorithms can be neutral, objective and controllable.”

## Privacy

Which definitions of data privacy need to be employed in particular circumstances of SD generation and use, and who creates these definitions? How can meaningful consent be obtained from individuals and communities when using their data to generate synthetic datasets? What notions of data ownership should pertain to the creation of SD?

AI ethics guidelines present privacy “both as a value to uphold and as a right to be protected” (Jobin et al, 2019) by means of data security laws and technical solutions. As a proverbial silver-bullet, SD promises to protect against privacy harms and to shorten lengthy data privacy approvals and consent procedures, saving both time and money. However, sensitive information about individuals can still be inferred from SD, especially when combined with auxiliary datasets (Jordon et al, 2022). Mitigating against such dangers may lower the utility value of the SD, meaning a trade-off between privacy and fidelity, which needs to be critically assessed. However, it is difficult to quantitatively evaluate such trade-offs because of the challenge of predicting which relationships between different data points are being either preserved or suppressed (Stadler et al, 2022).

## Transparency

How well does SD capture certain phenomena, and what specific phenomena is it even striving to represent? When using ‘real’ data to generate SD, how exactly does SD deviate from that ‘real’ data? When it is used to mitigate biases, what considerations were made, and what exactly is being mitigated?

Transparency is regularly referred to as “efforts to increase explainability, interpretability or other acts of communication and disclosure” (Jobin et al, 2019). Typically, transparency focuses on explaining the specific methods used in AI applications, and this is no different when it comes to SD. The methods used to generate SD do not necessarily deviate from other AI methods. The descriptions of the ‘real’ phenomena that SD is striving to represent and an evaluation of the degree to which it is successful are crucial dimensions of explainability. These are necessary requirements if SD is to serve as a sufficient and appropriate substitute for the real phenomena.

Given the complex nature, diverse use cases, and potential scale of SD, however, it is important to identify how existing ideas on transparency in AI are reshaped in the context of its use. For example, when SD is recursively used to infer meaningful information about ‘real’ phenomena, it is insufficient to solely disclose the method that was used to generate it. Beyond openness about data creation and modification, there is a clear need to separate and label SD, so as to avoid contamination and bias caused by inadvertently re-using datasets in future data analysis. The use of SD risks exacerbating the so-called “curse of recursion” where there is a recurrent absorption of falsehoods in applications and statements (Shumailov et al, 2023). When the results of one SD analysis are used to formulate another analysis, it becomes increasingly difficult to grasp the net ‘real’ world effects of a synthetic dataset.

“… there is a clear need to separate and label SD, so as to avoid contamination and bias caused by inadvertently re-using datasets in future data analysis.”

At present, it is not yet common practice for data researchers to keep track of the use of SD, even though generating and using SD in combination with existing datasets is becoming increasingly easy (D’Amico et al, 2023). This rapid surge of SD-use and re-use may exacerbate biases and discrepancies, whilst making it increasingly difficult to trace these flaws. Currently, new techniques for documenting how a specific set of (synthetic) data has been created and used are being developed, such as standardised forms for recording metadata (such as a ‘datasheet’ that captures how a dataset was created) (Gebru et al, 2021), or ‘watermarks’ for medical images (Qasim et al, 2018). These methods are essential to assess if and how SD has been used to make a calculation, and ultimately to evaluate if specific data analysis may result in unfair and unjust interpretations.

## Justice, fairness, and equity

When SD is used to improve representation, are diversity and emerging novelties within the underrepresented group adequately taken into consideration? How are developers remaining attentive to the emergence of new characteristics, traits or phenomena within the synthetic dataset? How can developers be alert to overreliance on SD in groups or populations where data collection is more difficult or costly?

In the context of AI ethics, justice is most frequently understood in terms of fairness, as well as the “prevention, monitoring or mitigation of unwanted bias and discrimination” (Jobin et al, 2019). Just AI requires inclusion and equity in creation, access and benefits of it, as well as access to redress in cases of potential harm. SD is often presented as a promising tool to reduce knowledge or training gaps related to underrepresented groups thereby achieving diversification. However, SD is not a magic fix to ensure equitable results. First, as previous research on ‘imputing’ missing racial and ethnical data shows, this adding of data implies an estimation of levels of variability that must be critically scrutinised for its consequences (Randall et al, 2021). Second, ‘de-biased’ SD could still lead to unjust outcomes as part of a broader discriminatory system, for example, when a risk score is subsequently implemented in an unfair way (DeCamp and Lindvall, 2023).

## Conclusion

Though this discussion is far from exhaustive, we have suggested that the redistribution of responsibilities, the reality gap, opaque trade-offs between privacy and fidelity, the reappraisal of transparency and the myth of reduced bias are just a few examples of the challenges we must consider when it comes to the future use(s) of SD. Beyond sharpening a critical perspective on SD, another significant ethical issue is how SD influences incentives and ideologies of research more broadly. SD is portrayed as a medium that greatly advances data-driven decision-making by mitigating privacy concerns and bias. However, the enthusiasm surrounding SD may serve to enlarge the incentive gap where we fail to appropriately recognise and reward “data work”, that is, the work that goes into collecting, curating and maintaining datasets (Gero et al, 2023).

The use of SD may also serve to amplify the prominence of using quantitative big-data research as a way of understanding and managing complex societal problems. A new focus on SD should not downplay the importance of other methods and frameworks, such as small-scale empirical data collection and qualitative research. The aim of SD generation is to enhance the capabilities of data-driven tools to improve well-being. For this to become a reality, questions about data ethics and justice must take a more prominent place in the generation and use of SD.

“A new focus on SD should not downplay the importance of other methods and frameworks, such as small-scale empirical data collection and qualitative research.”

### Supplementary information

Peer Review File
