# Supplementary material for: Getting real about synthetic data ethics: Are AI ethics principles a good starting point for synthetic data ethics?
Source: EMBO Rep. 2024 Feb 22;25(5):2152–5. doi: 10.1038/s44319-024-00101-0 (PMC11094102; doi:10.1038/s44319-024-00101-0)
Supplement: Supplementary file 1 — Peer Review File [file 44319_2024_101_MOESM1_ESM.pdf]

EMBO reports

Getting real about synthetic data ethics

Danielle Shanley, Joshi Hogenboom, Flora Lysen, Leonard Wee, Aiara Lobo Gomes, Andre Dekker, and Darian Meacham

Corresponding author(s): Danielle Shanley (danielle.shanley@maastrichtuniversity.nl)

Review Timeline:

Submission Date:	16th Jan 24
Accepted:	13th Feb 24

Editor: Holger Breithaupt

Transaction Report: This article was editorially reviewed.
